# Association Between the Risk of Hyperuricemia and Changes in Branched-Chain Amino Acids Intake Over Twelve Years: A Latent Class Trajectory Analysis From the China Health and Nutrition Survey, 1997–2009

**DOI:** 10.3389/fnut.2022.916446

**Published:** 2022-08-11

**Authors:** Xiyun Ren, Shasha Wu, Wei Xie, Ying Liu, Shucai Yang

**Affiliations:** ^1^Experimental Center for Preventive Medicine Teaching, College of Public Health, Harbin Medical University, Harbin, China; ^2^Department of Nutrition and Food Hygiene, College of Public Health, Harbin Medical University, Harbin, China; ^3^Translational Medicine Research and Cooperation Center of Northern China, Heilongjiang Academy of Medical Sciences, Harbin, China

**Keywords:** branched chain amino acids, hyperuricemia, latent class trajectory model, China health and nutrition survey, mediation analysis

## Abstract

**Objective:**

This study aims to identify dietary branched-chain amino acids (BCAA) consumption trajectories in Chinese adults and to evaluate their association with the risk of hyperuricemia (HU).

**Methods:**

Cohort data from the China Health and Nutrition Survey 1997–2009 were adopted in this research. A total of 6,810 participants aged ≥18 years were included in this study. Participants were designated into four subgroups on basis of the trajectories of dietary BCAA consumption. Cox proportional hazards models were performed to discuss the relationships between varied trajectories and the risk of HU after adjusting potential confounders. The intermediary effect of differential blood indexes between the trajectories and the risk of HU was explored with mediation analysis.

**Results:**

Four distinct trajectory groups of dietary BCAA consumption were identified. Compared with the low stable trajectory group, high to low trajectory group was greatly related to an increased risk of HU (HR 1.35 (95% CI 1.03 to 1.79)) with modification for covariates. Total cholesterol (TC), hemoglobin A1c (HbA1c), fasting blood glucose (FBG), and triglyceride (TG) partially regulated trajectories and HU.

**Conclusion:**

Gradually decreasing dietary BCAA intake increased the risk of HU, which is, at least, partially mediated by TC, HbA1c, FBG, and TG levels.

## Introduction

Hyperuricemia (HU) is a disease, in which serum uric acid (UA) exceeds the normal range due to abnormal purine metabolism for various reasons (1). The pooled prevalence of HU was 13.3% in mainland China from 2000 to 2014 (2). HU is considered a risk factor for gout (3), cardiovascular disease (4), stroke (5), diabetes (6), and hypertension (7). HU is becoming a great health issue and is arousing more attention. Currently, most studies on HU have been limited to some regions or nationality, and the pathophysiology of HU has not yet been fully illustrated. Hence, nationally representative research based on the entire population on the epidemiology of HU is needed.

Dietary factor, which is an adjustable element, exerts significant effect on the occurrence and development of HU. Branched-chain amino acids (BCAAs), including Leucine, Isoleucine, and Valine, an important group of basic amino acids, are significant nutrition signals with important roles in protein synthesis, glucose homeostasis, and nutrient-sensitive signaling pathways (8). Previous research has shown that adequate BCAA supplementation can reduce body weight and promote fat metabolism (9, 10). Meanwhile, BCAA can also reduce oxidative stress by restoring mitochondrial function (11). Overweight, obesity, abnormal lipid metabolism, and oxidative stress are potential risk factors for HU (12). However, the association between dietary BCAA consumption and risk of HU are still unknown. Most studies about dietary BCAA intake adopted a single or limited number of measurements. However, little research has been designed to investigate the dynamic change of dietary BCAA levels and the risk of HU. It is necessary to use a time-varying measurement of dietary BCAA to explore the relationship between dietary BCAA trajectory and HU risk.

Therefore, this study firstly used latent class trajectory modeling (LCTM) to identify dietary BCAA consumption trajectories over 12 years in the Chinese adults and to evaluate their association with the risk of HU.

## Materials and Methods

### Study Population

China Health and Nutrition Survey (CHNS), which stands for 47% of the Chinese population, is designed as prospective household-based research, including various ages and cohorts across different provinces, and five follow-up surveys between 1997 and 2009. Detailed information of CHNS has previously been provided (13). The Institutional Review Committees of the University of North Carolina at Chapel Hill, NC, United States approved the survey protocols, instruments, and process for acquiring informed consent, as well as the China National Institute of Nutrition and Food Safety at the Chinese Center for Disease Control and Prevention, Beijing, China. All participants offered survey data after written informed consent.

A total of 13,575 adults participated in CHNS from 1997 to 2009, with no missing value for BCAA intake were selected in this study. Individuals with missing serum UA information (*n* = 1,419) and data of demographic or total nutrient intakes dietary interview (n = 228) were excluded. Participates who took part in only one survey (*n* = 5,118) were excluded. After exclusion, a total of 6,810 adults, including 3,212 men and 3,598 women, with a mean age of 42.5 ± 13.4 years and a mean follow-up time of 9.98 years, met the study criteria and ranged from two to six measurement surveys (two visits, *n* = 807; three visits, *n* = 1,148; four visits, *n* = 1,550; five visits, *n* = 3,305).

### Questionnaire Survey

Detailed in-person interviews were managed by trained personnel with a structured questionnaire, to collect data of demographic features, dietary habits, lifestyle, physical condition, and anthropometric features. In CHNS, the collection of individual dietary intake for three consecutive days was made for every household member. Dietary measurements included total energy (kcal/day), dietary fat (g/day), dietary protein (g/day), dietary carbohydrate (g/day), dietary fiber (g/day), and vitamin C (VC, mg/day), which were calculated by three versions of Chinese food composition table (FCT) according to CHNS project requirements. The 1991 FCT version was adopted in 1997 and 2000. The 2002/2004 (two books integrated) FCT versions were adopted in 2004, 2006, and 2009. Present smoking was defined as a positive answer to the question “do you still smoke now?”. Participants who gave the answer of “never smoker” to the question “Have you ever smoked (such as hand-rolled or device-rolled)?” were categorized into the group of never smoked, and who positively answered the questions “Have you ever smoked (such as hand-rolled or device-rolled)?” and negatively answered “do you still smoke now?” as ex-smoker. Drinks and a standard drink was any drink that contained about 0.6 fluid ounces or 14 grams of pure alcohol were adopted to measure the amount of alcohol consumed (14). Physical activity level (PAL) mainly contained occupational activity, transportation activity, domestic activity, and leisure activity (15). The calculation of total metabolic equivalents (METs) of physical activity MET-h per week was made (15).

In 2009, 12 ml of blood (in three 4-ml tubes) were collected from all individuals on an empty stomach. The measurement of serum UA and fasting blood glucose (FBG) was made by enzymatic colorimetric and glucose oxidase (GOD-PAP) method (Hitachi 7600, Randox, United Kingdom). Total cholesterol (TC) and triglyceride (TG) were measured by cholesterol oxidase (CHOD-PAP) method and glycerol-phosphate oxidase (GPO-PAP) method (Hitachi 7600, Kyowa, Japan). The measurement of HDL-cholesterol (HDL-C) was made by enzymatic approaches (Hitachi 7600, Kyowa, Japan). The measurement of hemoglobin A1c (HbA1c) and high-sensitivity C reactive protein (hs-CRP) was made by high performance liquid chromatography method (HLC-723 G7/D10/PDQ A1c, Japan) and immunoturbidimetric (Hitachi 7600, Denka Seiken, Japan). HU was defined as serum UA ≥7 mg/dl in males and ≥6 mg/dl in females. Hypertension was defined as persistent systolic blood pressure measurements of ≥140 mmHg and/or 90 mmHg of diastolic blood pressure. The calculation of body mass index (BMI) as weight in kilograms divided by the square of height in meters was made. Self-report of a history of diabetes diagnosis, and/or FBG ≥ 7 mmol/L, and/or HbA1c ≥40 mmol/mol (6.5%), and/or receiving treatment for diabetes were adopted to identify Type 2 diabetes (T2D). Details of treating diabetes included special diet, weight control, oral medication, insulin injection, Chinese traditional medicine, and home remedies.

### Statistical Analysis

All statistical analyses were conducted with R 4.1.3^1^. A two-sided *p*-value <0.05 was of statistical significance. Dietary nutrient intake and anthropometric measurements were shown as mean ± standard deviation (SD) for continuous variables and percentage for categorical variables. General linear models and baseline features were compared with Chi-square tests.

The LCTM is a relatively new methodology in epidemiology to describe life-course exposures, which simplifies heterogeneous populations into homogeneous patterns or classes. Model fit was assessed using both the Bayesian information criterion and entropy measures in conjunction with Vuong-Lo-Mendell-Rubin likelihood ratio test. The LCTM has three general advantages. First, it better informs etiological associations by deeply phenotyping certain “at risk” subpopulations. Second, LCTM offers a public health strategy to identify early divergent adverse trajectories as potential intervention targets. Third, the trajectory approach allows a better understanding of the causes of between-individual variation in certain features.

Dietary BCAA consumption trajectories were identified with LCTM, a censored normal model applying the R package lcmm. The best fit and each trajectory class included at least 3% of the sample population, which were decided with statistically rigorous Bayesian information criteria. Once trajectories of dietary BCAA consumption were decided, and a nominal categorical variable was generated to illustrate the trajectory classes of every participant, which was then adopted in Cox multivariate regression models.

After calculating the follow-up time of non-HU and HU, Cox proportional-hazards model was adopted to assess the relationship between dietary BCAA consumption trajectories and the risk of HU. A set of potential confounders and effect modifiers, which were age, sex, smoking, education, PAL, urban index, total dietary energy, fat, protein, carbohydrate, VC intake, BMI, and disease state of hypertension and T2D, were controlled.

After categorizing participants into varied dietary BCAA consumption trajectories, the association between acquired dietary BCAA consumption trajectories and blood indexes modified with the above co-variables was determined with subgroup analyses by generalized linear models, which could recognize HU-associated blood indexes that were statistically different in varied trajectories.

At last, the R package lavaan was adopted to make mediation analysis models, to investigate whether these biomarkers with modification for the above covariates were adopted to mediate the relationship between dietary BCAA consumption trajectories and risk of HU.

### Sensitivity Analysis

Two sets of sensitivity analyses were made below: in set 1, blood samples from the participants were first collected in 2009, the relationship between dietary BCAA intake and the risk of HU for participants in 2009 was analyzed using logistic regression model; and in set 2, the association between mean dietary BCAA intake during follow-ups and the risk of HU from 1997 to 2009 was analyzed with logistic regression models.

## Results

### Trajectories of Dietary Branched-Chain Amino Acids Intake

**FIGURE 1:**
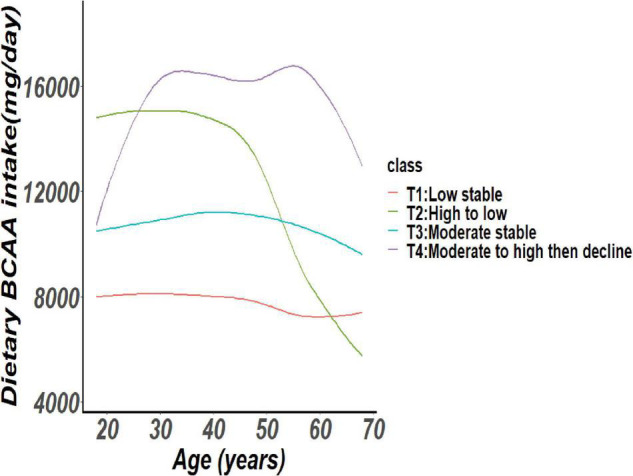
Trajectories of dietary branched-chain amino acids (BCAA) consumption (n = 6,810) in the Chinese adults from the CHNS by LCTM. BCAA, Branched chain amino acids; CHNS, China Health and Nutrition Survey; LCTM, Latent class trajectory modelling.

In this cohort of 6,810 Chinese adults, the trajectories of BCAA intake were displayed in Figure 1. Every trajectory group was named based on dynamic variations of the BCAA intake levels. Figure 1 corresponded with the first trajectory, labeled “T1: Low stable,” to participants who kept low BCAA intake throughout the survey period. The second trajectory, “T2: High to low,” corresponded to participants whose BCAA intake gradually decreased from high to low by comparing with T1. The third trajectory, “T3: Moderate stable,” corresponded to participants whose BCAA intake remained in moderate level by comparing with T1. The fourth trajectory, “T4: Moderate to high then decline,” corresponded to participants whose intake of BCAA gradually increased to moderate level and then decreases. It was estimated that the trajectories from T1 to T4 include 12.1, 7.8, 76.1, and 4% of participants.

### Baseline Features by Varied Trajectories of Dietary Branched-Chain Amino Acids Intake

The baseline characteristics of different dietary BCAA intake trajectories were displayed in Table 1. There were great diversities in age, gender, smoking, drinks, education levels, total energy, fat, protein, carbohydrate, VC intake, BMI, T2D, and hypertension among the four trajectories (*p* < 0.05). There was no great diversity in PAL among the different dietary BCAA intake trajectories (*p* > 0.05).

**TABLE 1:** Baseline characteristics of study variables by different trajectories of dietary branched-chain amino acids (BCAA) consumption (*N* = 6,810).

Trajectory	Low stable	High to low	Moderate stable	Moderate to high then decline	*P*-value
	(*n* = 821)	(*n* = 528)	(*n* = 5,188)	(*n* = 273)	
Age (years)	40.7(10.4)	34.4(14.8)	43.6(13.5)	41.9(8.0)	<0.001
Men (*n*,%)	157(19.1)	344(65.2)	2505(48.3)	206(73.8)	<0.001
High school education or above (*n*,%)	110(13.4)	126(23.9)	1,035(19.9)	59(21.1)	<0.001
Hypertension (*n*,%)	127(15.5)	77(14.6)	968(18.7)	48(17.2)	0.026
Current smoking (*n*,%)	152(18.5)	207(39.2)	1,620(31.2)	134(48.0)	<0.001
T2D (*n*,%)	117(14.2)	72(13.6)	977(18.8)	60(21.5)	<0.001
Total energy intake (kcal/day)	1,976.4(594.9)	2,692.2(884.8)	2,295.5(627.6)	2,764.9(991.3)	<0.001
Total fat intake (g/day)	53.7(28.2)	78.4(41.4)	68.9(35.7)	77.2(38.7)	<0.001
Total carbohydrate intake (g/day)	319.5(118)	411.7(150.5)	353.4(122.3)	424.5(173.5)	<0.001
Total protein intake (g/day)	55.6(33.3)	88.6(63.2)	68.5(24.2)	94.3(81.2)	<0.001
Total VC intake (mg/day)	81.4(53.1)	99.3(86.5)	88.3(70.5)	96.5(62.3)	0.033
Drinking (Drinks/week)	2.1(7.9)	5(10.6)	5.1(13.6)	8.5(15.7)	<0.001
Uric acid (mg/dL)	4.6(1.5)	5.5(1.8)	5.2(1.8)	5.6(1.7)	<0.001
Urban index	50.3(17.9)	54.6(20.5)	55.8(19.4)	54.9(20.5)	<0.001
BMI (kg/m^2^)	22.5(3.2)	22.8(3.0)	22.7(3.2)	22.3(3.0)	<0.001
PAL (Mets-h/week)	63.1(98.5)	85.5(116.7)	68.4(100.5)	95.1(120)	0.060

*Continuous data are expressed as mean (SD); where shown, data are n (%); SD, standard deviation.*

*Generalized linear models and Chi-square test were used to probe for differences in continuous variables and dichotomous variables.*

*PAL, Physical activity level; VC, Vitamin C; T2D, Type 2 diabetes; BMI, Body mass index.*

### Association Between Dietary Branched-Chain Amino Acids Intake Trajectories and Risk of Hyperuricemia

The relationships between dietary BCAA intake trajectories and risk of HU were displayed in Table 2. By comparing with “T1,” the trajectory labeled “T2” was greatly related to growing risk of HU (HR 1.35 (95% CI 1.03, 1.79)) with adjustment for covariates.

**TABLE 2:** Association between dietary branched-chain amino acids (BCAA) consumption trajectories and hyperuricemia (HU) by Cox regression models.

Trajectory	Case/*N*	Model 1	Model 2	Model 3
		HR (95% CI)	HR (95% CI)	HR (95% CI)
Low stable (T1)	118/821	1(Ref.)	1(Ref.)	1(Ref.)
High to low (T2)	97/528	1.43(1.09,1.87)	1.39(1.06,1.83)	1.35(1.03,1.79)
Moderate stable (T3)	974/5,188	1.19(0.84,0.98)	1.17(0.96,1.42)	1.14(0.94,1.39)
Moderate to high then decline (T4)	61/273	1.25(0.80,0.91)	1.24(0.90,1.70)	1.20(0.88,1.66)
*P* for trend		0.540	0.710	0.800

*Model 1 Adjusted for age, sex, smoking, drinking, education, urban index, and PAL. Model 2 was further adjusted by total energy, fat, protein, carbohydrate, and VC intake.*

*Model 3 was further adjusted by BMI, hypertension, and T2D status.*

*PAL, Physical activity level; VC, Vitamin C; T2D, Type 2 diabetes; BMI, Body mass index.*

### Trajectories of Dietary Branched-Chain Amino Acids Intake and Biomarkers of Hyperuricemia

The biomarkers across different dietary BCAA intake trajectories were shown in Table 3. TG, TC, FBG, and HbA1c in the T2 trajectory were higher than the other three trajectories (T1, T3, and T4) (all *p* for trend <0.05). HDL-C and hs-CRP in the T2 trajectory were not statistically different from the other trajectories.

**TABLE 3:** Difference for hyperuricemia (HU)-related factors across dietary branched-chain amino acids (BCAA) consumption trajectories in 2009.

Variables	T1	T2	T3	T4	*P*-value
HDL-C (mmol/L)	1.43(0.38)	1.41(0.54)	1.45(0.49)	1.42(0.34)	0.232
hs-CRP (mg/L)	2.90(13.19)	3.44(22.77)	2.61(6.43)	2.43(5.05)	0.177
TC (mmol/L)	4.83(0.99)	4.94(0.91)	4.74(1.01)	4.92(0.97)	< 0.001
TG(mmol/L)	1.64(1.39)	1.75(1.50)	1.73(1.31)	1.68(1.50)	0.007
FBG (mmol/L)	5.32(1.41)	5.47 (1.38)	5.34(1.50)	5.44(1.21)	0.004
HbA1c (%)	5.58(0.79)	5.69(0.82)	5.66(0.95)	5.58(0.82)	0.005

*Generalized linear model was used to probe for the HU-related factors differences across different trajectories. Data are mean (SD).*

*HDL-C, High density lipoprotein cholesterol; TC, Total cholesterol; TG, triacylglycerol; hs-CRP, High sensitivity C reactive protein; HbA1c, Hemoglobin A1c FBG, Fasting blood glucose; SD, standard deviation.*

### Mediation Analysis

**FIGURE 2:**
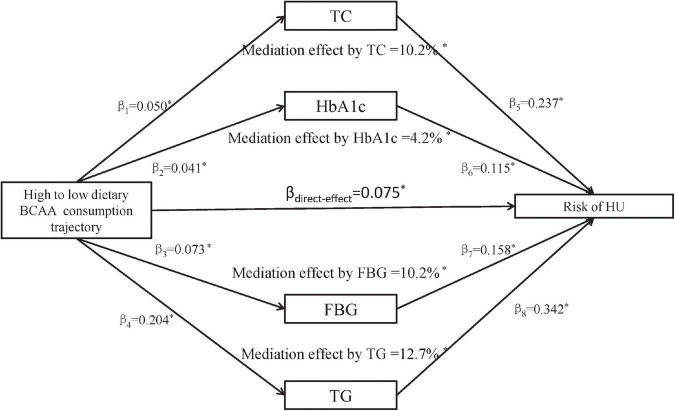
Mediation effects of TC, HbA1c, FBG, and TG on the association between dietary branched-chain amino acids (BCAA) consumption trajectories and risk of HU. Data were standardized regression coefficients with adjustment for covariates; *p < 0.05 for coefficients different from 0. TC, Total cholesterol; HbA1c, Hemoglobin A1c; FBG, Fasting blood glucose; TG, Triglyceride; BCAA, Branched chain amino acids; HU, Hyperuricemia.

Figure 2 demonstrates mediation results of TC, HbA1c, FBG, and TG in the relationship between dietary BCAA trajectory (T2) and risk of HU. The total effect of dietary BCAA intake trajectories was estimated at 7.5%. The total indirect role for four factors was calculated with β1 to β8. It was estimated that the percentages of the total effect mediated by TC, HbA1c, FBG, and TG are 10.2%, 4.2%, 10.2%, and 12.7%, respectively.

### Sensitivity Analysis

Sensitivity analyses demonstrated that compared to the lowest quintile, the highest quintile of dietary BCAA intake was not greatly related to the risk of HU in 2009 (OR, 1.16, 95%CI (0.76, 1.33)) in Table 4. Similarly, the highest quintile of mean dietary BCAA intake during follow-ups was also not significantly associated with the risk of HU from 1997 to 2009 (OR, 1.18, 95%CI (0.94, 1.49)) in Table 5.

**TABLE 4:** Association between dietary branched-chain amino acids (BCAA) consumption and hyperuricemia (HU) by Logistic regression models in 2009 (*N* = 6,810).

BCAA intake	Q1 ≤ 7,579.6	Q2 (7,579.7-9,299.3)	Q3 (9,299.4-11,088.9)	Q4 (11,089.0-13,531.9)	Q5 ≥ 13,531.9	*P* for trend
Case/N	222/1,362	249/1,362	227/1,362	273/1,362	279/1,362	
Model 1	1(Ref.)	1.16(0.93,1.45)	1.04(0.81,1.33)	1.31(0.98,1.76)	1.32(0.88,2.00)	0.150
Model 2	1(Ref.)	1.17(0.96,1.44)	1.04(0.84,1.28)	1.33(1.09,1.39)	1.37(1.12,1.68)	0.001
Model 3	1(Ref.)	1.11(0.89,1.39)	1.00(0.78,1.30)	1.21(0.90,1.63)	1.16(0.76,1.33)	0.390

*Model 1 Adjusted for age, sex, smoking, drinking, education, urban index, and PAL.*

*Model 2 was further adjusted by total energy, fat, protein, carbohydrate, and VC intake.*

*Model 3 was further adjusted by BMI, hypertension and T2D status.*

*PAL, Physical activity level; VC, Vitamin C; T2D, Type 2 diabetes; BMI, Body mass index.*

**TABLE 5:** Association between Mean dietary branched-chain amino acids (BCAA) intake during follow-ups and hyperuricemia (HU) by Logistic regression model (*N* = 6,810).

BCAA intake	Q1 ≤ 6,203.3	Q2 (6,203.4-8,172.8)	Q3 (8,172.9-9,674.6)	Q4 (9,674.7-11,324.6)	Q5 ≥ 11,324.6	*P* for trend
Case/*N*	241/1,362	234/1,362	254/1,362	256/1,362	265/1,362	
Model 1	1(Ref.)	0.96(0.77,1.19)	1.15(0.93,1.43)	1.12(0.90,1.39)	1.24(0.99,1.39)	0.020
Model 2	1(Ref.)	0.98(0.80,1.21)	1.16(0.95,1.42)	1.18(0.96,1.44)	1.30(1.06,1.60)	0.002
Model 3	1(Ref.)	0.92(0.73,1.15)	1.00(0.78,1.30)	1.12(0.90,1.40)	1.18(0.94,1.49)	0.060

*Model 1 Adjusted for age, sex, smoking, drinking, education, urban index, and PAL.*

*Model 2 was further adjusted by total energy, fat, protein, carbohydrate, and VC intake.*

*Model 3 was further adjusted by BMI, hypertension and T2D status.*

*PAL, Physical activity level; VC, Vitamin C; T2D, Type 2 diabetes; BMI, Body mass index.*

## Discussion

In this prospective cohort of Chinese adults with five surveys, four unique dietary BCAA intake trajectories were identified, in which the high to low trajectory group was greatly related to increased risk of HU. In addition, high to low trajectory has higher TC, HbA1c, FBG, and TG than other trajectories, which may explain the association between trajectory and HU.

Obesity is considered as an independent risk factor for HU. Obesity can cause HU by increasing UA synthesis and inhibiting its excretion. Changes in obesity measure indices levels are independently associated with subsequent changes in UA concentrations. Previous studies have shown a strong association between obesity and HU (16–18). Higher dietary BCAA intake was related to lower prevalence of overweight and obesity in Asia (19). BCAA contributes to the oxidation of fat, and decreases fat in the body (20). Increased dietary leucine intake significantly reduced body weight and improved glucose and cholesterol metabolism (21). It has been found that adenosine monophosphate-activated protein kinase (AMPK) is involved in the basic control of whole-body energy balance by controlling food intake and energy expenditure in response to hormonal and nutritional signals in the central nervous system and peripheral tissues (22). Negative AMPK expression in the hypothalamus is clearly enough to decrease food intake and body weight (23). Mammalian target of rapamycin (mTOR), which is a serine/threonine kinase, takes part in a lot of cellular processes, such as protein synthesis, cell metabolism, and growth (24). Studies have shown that activation of mTOR can inhibit food intake (25). However, dietary leucine intake can lead to weight loss by reducing AMPK or increasing mTOR activity (26). Meanwhile, the BMI of the high to low trajectory group was greatly higher than the other trajectory groups in this research, which confirmed the results of previous studies.

The systemic inflammatory response index (SIRI), a potent indicator of HU, is independently associated with the risk of HU (27). Studies have also shown that decreasing the intake of proinflammatory diet decreases the incidence of HU in women (28). However, BCAA plays an anti-inflammatory role in the body or indirectly regulate**s** inflammatory states (29). Reducing the intake of BCAA may increase inflammation in the body and lead to the occurrence of HU. The hs-CRP is widely used laboratory markers of systemic inflammation. Previous studies demonstrated no correlation between hs-CRP levels and valine (30), and there was no statistical difference in hs-CRP between different trajectories in this study, which was consistent with previous research result.

Insulin resistance (IR) is closely associated with the occurrence and growth of HU by inhibiting UA excretion and increasing sodium reabsorption in renal tubules (31, 32). It is well-known that increased glucose content in the liver is a major characteristic of IR. Elevated liver glucose content is due to the activation of c-Jun N-terminal kinase (JNK) caused by the produced reactive oxygen species (ROS), leading to phosphorylation of FoxO1 and nuclear accumulation of FoxO1, and ultimately leading to increased liver glucose content (33). Intake of BCAA can improve IR by activating antioxidant mechanisms and can reduce ROS production in the liver by improving albumin metabolism (34). A cross-sectional study has demonstrated that higher intakes of BCAA were associated with lower IR, inflammation, blood pressure, and adiposity-related metabolites (35). Therefore, the risk of HU might be reduced by BCAA intake.

Blood glucose control is of great significance for the prevention of HU (36). Studies have shown that increased isoleucine intake can stimulate the uptake of glucose by skeletal muscle, leading to a reduction in blood glucose (37). Growing leucine intake can enhance glucose metabolism, decrease the insulin resistance induced by diets, and lower levels of plasma glucagon and the expression of hepatic glucose 6 phosphatase, which is the key enzyme for regulating hepatic glucose production (38). In addition, previous studies have shown that UA levels increase significantly with the increase of FBG in the non-diabetic phase (39). Leucine levels were inversely correlated with FBG, and FBG increased when leucine intake was reduced (40). Reduced BCAA intake will lead to increased FBG, which can increase UA levels in the body.

The alteration of blood lipid may be another mechanism to explain our observations. Higher TG and TC levels were significantly correlated with the increase of serum UA (41). The risk of HU in patients with hypertriglyceridemia was higher than those in patients with normal TG (42). High TG may disrupt the metabolism of free fatty acids and accelerate the breakdown of adenosine triphosphate, which ultimately results in a growth in UA (43). Isoleucine reduces TG accumulation by affecting fatty acid oxidation (44), which decreases the incidence of HU. Animal studies have shown that compared to the control group, when mice consumed more isoleucine, the adiposity of liver and skeletal muscle was less, which indicated that the TG levels in both tissues were lowered. Several studies have shown that higher intake of BCAA presents lower occurrence of hypertriglyceridemia (45).

There are several strengths in this study. Primarily, this is the first research in this subject area that was made with a relatively great cohort size and long follow-up duration. Secondly, this research firstly explored the association between the dynamic trajectory of dietary BCAA intake and the risk of HU by using LCTM in Chinese adults. Thirdly, results of this study using dietary trajectory analysis were not presented in cross-sectional data, which meant that the study using dietary trajectory method to discuss the association between dietary BCAA intake, and the incidence of HU might provide additional information in cohort study and emphasized the significance of utilizing LCTM to scientific research. Nevertheless, it was recognized that there were several restrictions in this research. First, the dietary data were acquired by questionnaires in CHNS, and the respondents might have misreported the mount and kinds of food intake, leading to inaccurate mean value for BCAA measurement in three consecutive days. Secondly, several confounding elements, which were not measured or recognized, might have affected the outcomes.

## Conclusion

Gradually decreasing dietary BCAA intake increased the risk of HU, which is, at least, partially mediated by TC, HbA1c, FBG, and TG levels.

## Data Availability Statement

The original contributions presented in this study are included in the article/supplementary material, further inquiries can be directed to the corresponding author/s.

## Author Contributions

YL and SY conceived the concept. XR designed the research and prepared the original manuscript. WX researched and explained data. SW made the validation analyses. All authors have read and agreed to the published version of the manuscript.

## Footnotes

1
www.r-project.org/
